# Prevention of Low Back Pain in Adults with a Back School-Based Intervention

**DOI:** 10.3390/jcm10225367

**Published:** 2021-11-18

**Authors:** Pablo Hernandez-Lucas, Juan Lopez-Barreiro, Jose Luis Garcia-Soidan, Vicente Romo-Perez

**Affiliations:** Faculty of Education and Sport Sciences, Universidade de Vigo, Campus a Xunqueira, s/n., 36005 Pontevedra, Spain; juan.lopez.barreiro@uvigo.es (J.L.-B.); jlsoidan@uvigo.es (J.L.G.-S.); vicente@uvigo.es (V.R.-P.)

**Keywords:** exercise, educational intervention, primary prevention, musculoskeletal pain, rehabilitation

## Abstract

Background: Low back pain is highly prevalent and has a major socio-economic impact worldwide. Among the rehabilitation options is the Back School, which consists of programmes that include exercise and educational interventions to treat and prevent back pain. The effects of this type of programme are usually evaluated in patients with low back pain. The aim of this study was to evaluate the effects on low back functionality and the prevention of medical visits due to low back pain during one year of follow-up in a healthy adult population. Methods: A quasi-experimental study was conducted with 56 healthy participants who were divided into an experimental group (*n* = 30), who underwent the programme consisting of a total of 16 sessions, and a control group (*n* = 26), who did not undergo the intervention. All participants were administered the Partial Curl-up Test, Biering Sorense Test, Modified Schöber Test, and Toe Touch Test, and they completed the Short Form 36 Health Survey before and after the intervention. In addition, a telephone call was made to ask whether they attended a doctor for low back pain in the following year post-intervention. Results: In the experimental group, statistically significant improvements were observed in trunk muscle strength, spinal flexion joint range of motion, and hamstring flexibility, and they had fewer visits to the doctor for low back pain in the following year. Conclusions: The theoretical–practical programme based on the Back School seems to have beneficial effects on low back functionality by increasing its strength and flexibility in an adult population. In addition, this programme reduced the number of medical visits due to low back pain during the following year after the intervention.

## 1. Introduction

Low back pain (LBP) is a common and disabling issue, ranked as the leading cause of years lived with disability in adults [1]. LBP has become one of the main causes of demand for medical care in developed countries and a major cause of incapacity for work, with a consequent economic cost [2,3,4].

It is essential to know the main risk factors associated with LBP in order to be able to act on them by way of prevention, with the objective of achieving a reduction in the serious socio-economic repercussions caused by LBP [5]. This condition has a multifactorial origin [6]: sedentary lifestyle [7], obesity [8], lack of trunk musculature strength [9,10], lack of flexibility [11,12], psychosocial [13], and work-related factors [14].

European clinical guidelines highlight the importance of exercise and educational interventions in the treatment and prevention of LBP [15]. The Back School (BS) is one of the most widely used therapeutic methods for the prevention of low back pain. BS consists of a theoretical–practical programme that aims to teach abilities that protect the health of the back to healthy people or people with lumbar pathology [16]. These biopsychosocial programmes transmit theoretical concepts of anatomy, healthy lifestyle recommendations, and information about erroneous beliefs regarding the causes or origin of LBP, and in the practical part, patients are taught to perform strengthening and stretching exercises for the back [16].

There is scientific evidence on the beneficial effects of BS in people with LBP: improved quality of life [17,18,19], decreased pain [17,18,19,20,21,22], and reduced disability [17,18,19,20,21,22,23]. However, there is limited evidence of the effects of BS on this region in healthy adults, despite the fact that one of the main goals of BS is primary prevention [16]. Therefore, the aim of this research was to investigate the effects of a BS-based intervention on low back functionality and prevention of medical visits during one year of follow-up in healthy adults. It was previously hypothesised that this BS-based intervention would have positive effects on low back functionality by increasing strength and flexibility in healthy adults and reducing the number of medical visits due to LBP.

## 2. Materials and Methods

### 2.1. Design

A quasi-experimental control group (CG) study was conducted using a convenience sample, in which scores on measures of the dependent variables were compared before and after the intervention, both in the experimental group (EG) (people who attended the Back School sessions) and in the CG (people who did not attend the Back School sessions) to compare the possible effects of the intervention. 

### 2.2. Participants

A non-probabilistic purposive sampling was carried out at the Municipal Sports Centre of Pontevedra (Spain). The following inclusion criteria were applied to 98 volunteers: (a) age between 18 and 65 years; (b) no regular physical activity in the last three months; (c) not taking medication or presenting any musculoskeletal, rheumatic, metabolic, cardiological or neurological disorder, or previous clinical history of LBP or back pathologies; (d) signing the informed consent form. The following exclusion criteria were also used: (a) missing more than two Back School sessions; (b) not being able to attend the measurement sessions; (c) varying their lifestyle in relation to physical activity, rest, and nutrition; (d) taking any medication or having any musculoskeletal, rheumatic, metabolic, cardiological, or neurological disorder.

**Figure 1:**
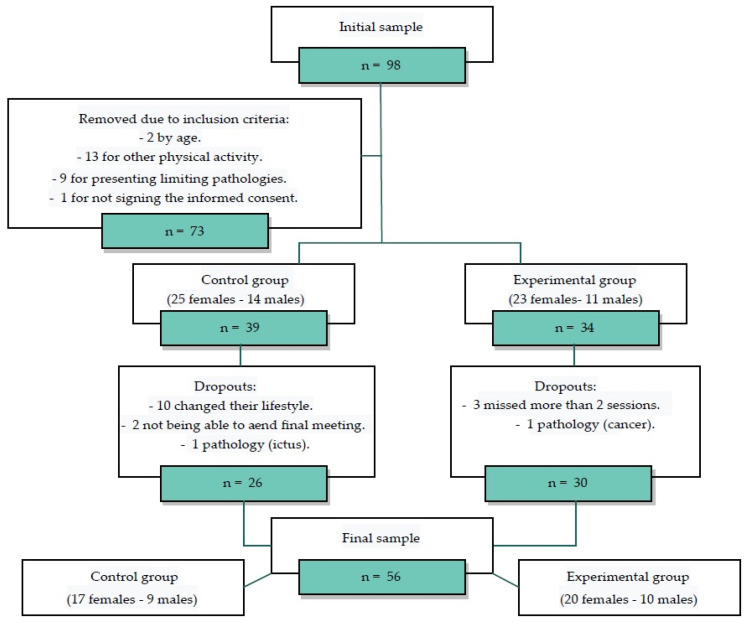
Sample selection flowchart.

The sample, after applying the inclusion and exclusion criteria, consisted of a total of 73 participants. Participants were assigned to the CG if they were unable to attend the timetable of the Back School sessions and to the EG if they attended the Back School sessions. During the study there were 17 dropouts: 13 from the CG and 4 from the EG. The final number of participants was 56 (Figure 1).

### 2.3. Intervention and Procedure

The study consisted of an intervention based on the Back School. This intervention was carried out for 8 weeks with a frequency of two sessions per week, with a total of 16 sessions lasting 45 min. Of all the sessions, 14 had a practical focus and the other two had a theoretical focus. In addition, they had an initial session and one at the end of the intervention in which measurements of the different variables were carried out. In the following year, they received a telephone call asking whether they had seen a doctor because of LBP. A summary of the intervention and procedure carried out in this study is shown in Table 1.

#### 2.3.1. Data Recording Protocol

Data recording was carried out at both the CG and the EG the day before the first session (pre-test) and the day after the last session (post-test). All measurements were performed by the same physiotherapist at a constant temperature of 20 degrees centigrade and between 10:00 and 12:00 a.m. Participants brought comfortable sports clothing.

The recording was carried out in the following order: (a) informed consent; (b) anthropometric measurements; (c) 7 min warm-up with vegetative activation through joint mobility exercises; (d) Partial Curl-Up Test; (e) Biering Sorensen Test; (f) Modified Schöber Test; (g) Toe Touch Test; (h) Short Form-36.

#### 2.3.2. Theoretical Sessions

The first session was given by a registered physiotherapist. This session aimed to teach, in an applied way, the anatomy and biomechanics of the spine and to inform participants about catastrophic misbeliefs regarding the causes and origin of LBP.

The second session was delivered via videoconference by a registered psychologist. This session aimed to teach, using images and examples, the main psychosocial factors that can influence the perception of pain such as emotions or previous experiences of pain. Stress management skills were also practised.

#### 2.3.3. Practical Sessions

The practical classes lasted 45 min. They were structured as follows: doubts, warm-up, main part, and cool-down.

In the first part of each class, participants were asked about their doubts and reviewed the principles of each exercise. This lasted for 3 min. In the sessions where there were no doubts, the physiotherapist asked questions about the content seen in the theoretical classes with the aim of recalling knowledge, thus integrating both parts: theory and practice.

In the warm-up, the participants performed active joint mobility exercises lasting two minutes in each of these areas of the body: lower limbs, upper limbs, and back. The warm-up ended with a 1 min walk alternating between walking on tiptoe and heels. In the main part, activation exercises of the trunk-stabilising muscles were intercalated with active breaks consisting of light stretching and joint mobility exercises. During the first five practical classes, the exercises were performed without implements (Table 2). In the next three sessions, a light resistance band was used as an implement. In sessions 12, 13, and 14 a half kilogram toning ball was used as an implement. In the last three classes a one-kilogram dumbbell was used as resistance. This part lasted 30 min.

In the cool down, the focus was on stretching, breathing, and relaxation exercises. This part lasted 5 min.

### 2.4. Study Variables

Information was recorded for all participants’ age, weight (using a scale (Tanita™ b303, Tokyo, Japan)), and height (using a homologated tallimeter (Seca™ 709, Hamburg, Germany)). Participants stood feet together, without shoes, with their head in the Frankfort plane.

#### 2.4.1. Strength of the Trunk Flexor Musculature

To measure the strength of the trunk flexor musculature, participants performed The Partial Curl-up Test (PCT). The test consists of performing the maximum number of curl-ups in one minute. The participant laid supine with their knees flexed at 90 degrees and their feet flat on the floor. The arms were extended along the trunk. Two lines were drawn, one at 8 cm (for participants over 40 years old) and one at 12 cm (for participants under 40 years old). The greater the number of repetitions, the greater the strength of the abdominal muscles [24].

#### 2.4.2. Strength of the Trunk Extensor Musculature

To measure the strength of the trunk extensor musculature, the Biering Sorense Test (BST) was performed. In the test, the participant was placed in prone position so that his or her anterior superior iliac spines coincided with the edge of the table, arms crossed over the chest. Participants were asked to keep their upper body in a horizontal position until they could no longer maintain the posture. The longer the participant held the position, the greater the strength of the trunk extensor musculature [24,25].

#### 2.4.3. Range of Motion of Back Flexion

To measure the variable range of motion (ROM) of back flexion, the Modified Schöber Test (MST) was used, which is considered a “Gold Standard” test for the measurement of lumbar flexion [26].

For the test, patients stood upright with their feet hip-width apart. Two lines were drawn, one 5 cm below the lumbosacral junction and the other 10 cm above. The patients then performed maximum trunk flexion without bending their knees and the distance between the marks was measured again [26].

#### 2.4.4. Hamstring Flexibility

To measure the variable hamstring flexibility, the Toe Touch Test (TTT) was used. To perform the test, the participant stood on a box with bare feet at shoulder width and performed a trunk flexion with their knees straight. The distance from the base of the box was measured in centimetres. Values above zero on the yardstick (coinciding with the support surface of the feet on the drawer) were considered negative, and those above zero below the yardstick, were considered positive. The register was recorded in centimetres [27].

#### 2.4.5. Health-Related Quality of Life

The Spanish version of the Short-Form Health Survey (SF-36) was used to measure quality of life [28].

The SF-36 explores people’s physical and mental health. It consists of 36 items that assessed eight dimensions of health status: social function, physical function, emotional role, physical role, mental health, vitality, physical pain, and general health. Scores ranged from 0 (worst health status) to 100 (best health status) [29].

#### 2.4.6. Medical Visits

To measure the dichotomous variable medical visits (MV), each participant was asked by telephone if they had seen a doctor due to LBP in the last year after undergoing this BS-based intervention.

### 2.5. Ethics

The study was evaluated and approved by the scientific committee of the Faculty of Education and Sports Sciences at the Universidade de Vigo, registration number 05-0721. The present study adhered to the ethical standards of the Declaration of Helsinki. In addition, all participants signed an informed consent form before the start of the study.

### 2.6. Statistics Analysis

The normal distribution of the data was verified using the Shapiro–Wilk test and homogeneity of variance with Levene’s test. Both pre-intervention groups were found to show no significant differences in the variables under study with the t-test for independent samples.

An ANOVA2x2 (Group × Time) analysis was used to analyse the effects of the intervention for all variables except for the dichotomous variable, MV, for which Fisher’s F-test was used.

Finally, effect size was calculated with the Cohen’s d statistic, defined as small: d = 0.1; medium: d = 0.5; large: d = 0.8 and the Cramer’s V test on the dichotomous variable, MV, defined as small: v = 0.1; medium: v = 0.3, large: v = 0.5 [30]. The significance level was set at *p* < 0.05. Analyses were performed with STATA 15.0 for MacOS^®^ software (STATA Corporation, College Station, TX, USA).

## 3. Results

In Table 3, the pre-intervention values are detailed. No significant differences were found in any of the variables analysed between the groups at baseline.

### 3.1. Trunk Flexor Musculature Strength Results

In the PCT, significant differences were found with respect to the factor Group *F*_(1-108)_ = 10.44, *p* = 0.01; the factor Momentum *F*_(1-108)_ = 4.81, *p* = 0.03; and the interaction (Group × Momentum) *F*_(1-108)_ = 4.3, *p* = 0.04 (Table 4). This was with an effect size of medium and a percentage improvement of 9.9% in the strength of the flexor musculature.

### 3.2. Trunk Extensor Musculature Strength Results

Significant differences were also found in the BST with respect to the factor Group *F*_(1-108)_ = 4.78, *p =* 0.03; the factor Momentum *F*_(1-108)_ = 3.99, *p* = 0.048; and the interaction (Group × Momentum) *F*_(1-108)_ = 6.75, *p* = 0.01 as shown in Table 4. This was with a medium effect size and percentage improvement of 10.5% in the extensor musculature strength.

### 3.3. Range of Motion Result of Back Flexion

As shown in Table 4, significant differences were found in the LST with respect to the factor Group *F*_(1-108)_ = 6.5, *p* = 0.01; factor Momentum *F*_(1-108)_ = 4.98, *p* = 0.03; and the interaction (Group × Momentum) *F*_(1-108)_ = 5.74, *p* = 0.02. This was with a large effect size and with a percentage improvement of 6.5% in ROM in flexion.

### 3.4. Hamstring Flexibility Result

In the TTT, as detailed in Table 4, with respect to the factor Group *F*_(1-108) =_ 3.42, *p* = 0.08, with respect to the factor Momentum *F*_(1-108) =_ 6.4, *p* = 0.01, and with respect to the interaction (Group × Momentum) *F*_(1-108)_ = 6.94, *p* = 0.01. This was found with an effect size of large and a percentage improvement of 452% in hamstring flexibility.

### 3.5. Health-Related Quality of Life Result

In the fSF-36, no significant differences were found in the physical dimension with respect to the factor Group *F*_(1-108)_ = 1.21, *p* = 2.73, nor with respect to the factor Momentum *F*_(1-108)_ = 0.25, *p* = 0.62, and neither with respect to the interaction (Group × Momentum) *F*_(1-108)_ = 1.09, *p* = 0.3.

In pSF-36 a significant difference was obtained with respect to the factor Group *F*_(1-108)_ = 5.64, *p* = 0.02, but not with respect to the factor Momentum *F*_(1-108)_ = 0.02, *p* = 0.89 or with respect to the interaction (Group x Momentum) *F*_(1-108)_ = 0.44, *p* = 0.51 (Table 4).

### 3.6. Result of Medical Visits

In the CG, four participants visited the doctor for LBP while in the EG no participants visited the doctor. After the intervention, the Fisher’s F-test for the dichotomous variable MV revealed a significant difference *p* = 0.04 and a medium effect size Cramer’s V = 0.30.

## 4. Discussion

The aim of the research was to determine the effects of a BS-based intervention on low back functionality in healthy adults and on the number of medical visits due to LBP during the following year after the intervention. The results of the study suggest that the effect is positive, including those obtained in both the strength of the trunk extensor musculature and the strength of the extensor musculature, as well as the improvement in the ROM of lumbar spine flexion and hamstring flexibility. Positive results were also observed in the number of medical visits due to LBP in the EG.

One of the main risk factors for LBP is the lack of strength in the trunk musculature [10]. In this study, after completing the BS programme, the strength of participants’ trunk flexor and extensor musculature increased. In the article by Behennaha et al. [9], he concludes that strengthening these musculatures can be helpful in the prevention of LBP. Another risk for LBP is lack of flexibility [11]. Participants in this study showed increased back flexion ROM and hamstring flexibility. Tousignant et al. [12] also found a relationship between low back pain and lumbar flexion ROM measured with the MST in their study. This same test was used in our study. Similarly, the article by França et al. [11] concludes that trunk stabilisation and stretching exercises improve functionality in patients with chronic non-specific LBP; these types of exercises are the ones used in this article. The study by Gül et al. [31] concludes that the combination of exercise and education is more effective in increasing abdominal strength and improving kinesophobia in LBP patients than only performing exercises. In all BS programmes, there is always a combination of exercise and education.

Psychosocial factors can also affect LBP [13,14]. A clear example of this is the anxiety generated in the patient by catastrophic misbeliefs or fear of the unknown. According to the definition of the International Association for the Study of Pain, LBP represents not only sensory awareness of physical damage but also an emotional experience that can be influenced by other emotions including anxiety or fear [32]. Therefore, in pain prevention, it is interesting to educate and inform the patient about the origins and causes of LBP. Paolucci et al. [19] concluded that BS improves mental state and quality of life. Again, in the results of the study of Morone et al. [21], quality of life was significantly improved more over time in BS in physical and mental score. However, the participants in this study did not find statistically significant improvements. This could be the result of differences in the sample between the articles, as the previous two articles had participants with LBP, and our sample was a healthy population without LBP. It is also worth noting that if we compare the mean values of the SF-36 survey of our study sample with the mean values of the Spanish population according to the study by Vilagut et al. [29], our sample has a 16% higher score than the Spanish mean. These high scores may have been the reason for not obtaining significant results in our sample population.

Considering the benefits obtained on the different risk factors for LBP and the significant reduction in the number of medical visits, BS is a programme that can help prevent LBP. Minghelli et al. [33] in their BS intervention, also with one year of follow-up, obtained positive results in the LBP perceived by their participants, although the sample of their study was made up of adolescents and not of adults as in this study. A systematic review of LBP clinical practice guidelines concluded that exercise and education are the most effective non-pharmacological therapies for the treatment and prevention of LBP. In addition, BS programmes are recommended in six of the eight guidelines reviewed in this study [34].

The limitations of the study include the dispersed age of the participants, which meant that the effect of aging was not considered, as well as not stratifying the results by age and gender due to the limited number of participants. In terms of future research, it would be interesting to use a larger sample size or to conduct research by age range and gender. It is also worth mentioning that there were no measurements of the research tools used after one year of the intervention except for the follow-up phone call. However, to our knowledge, this is the first study to analyse the number of visits to the doctor during one year of follow-up in healthy adults after participating in a BS intervention, which is an important parameter for assessing the efficacy in the prevention of LBP. Considering all the above, it would be interesting to carry out new studies with longer-term follow-up in healthy people and in specific populations with low back pain.

## 5. Conclusions

This BS-based intervention could improve low back functionality by increasing its strength and flexibility in an adult population. In addition, this programme reduces the number of medical visits due to LBP during the following year after the intervention. This programme could be implemented in gyms, primary care centers or companies for a low price, thus acting as a preventive measure and reducing the serious socio-economic repercussions caused by LBP. Further studies including longer-term follow-up are needed to validate these results.

## Figures and Tables

**Table 1:** Summary of the intervention and procedure.

N	Session Type	Name	Main Objective of the Session
1	Data registry	Pre-test registry	Obtain informed consent and initial sample measurements
2	Theory	Anatomy and LBP risk factors	Learn the basics of anatomy, biomechanics, and clarification of erroneous beliefs regarding the causes or origin of LPB
3–5	Practice	Exercises without implements.	Do and learn strength and flexibility exercises without implements
6	Theory	Psychosocial LBP risk factors	Learn about psychosocial LBP factors and to learn stress management techniques
7–8	Practice	Exercises without implements	Do and learn strength and flexibility exercises without implements
9–11	Practice	Exercises with elastic band	Do and learn strength and flexibility exercises with light resistance band
12–14	Practice	Exercises with Toning Ball	Do and learn strength and flexibility exercises with the 0.5 kg toning ball
15–17	Practice	Exercises with dumbbell	Do and learn strength and flexibility exercises with the 1 kg dumbbell
18	Data registry	Post-test registry	Obtain participants’ measurements at the end of the intervention
19	Data registry	Post-test phone call	Obtain number of medical visits due to LBP in the last year

**Table 2:** Overview of the exercises in the main part.

Name	Starting Position	End Position	Duration (min)
Squat	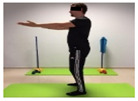	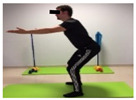	2
Isometric abdominal	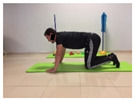	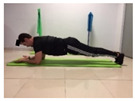	0.5
Alternate leg extension	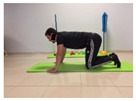	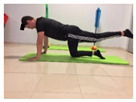	2
Isometric abdominal	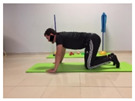	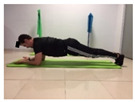	0.5
Alternative arm/leg extension	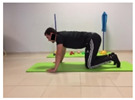	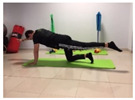	2
Lateral leg raises; change sides every 30 s	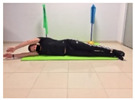	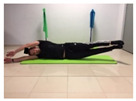	2
Lateral trunk raise; change sides every 30 s	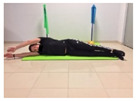	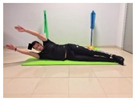	2
Arm/leg raise; change sides every 30 s	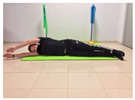	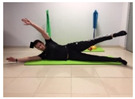	2
Shoulder bridge	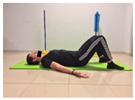	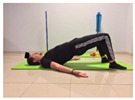	2
Abdominal exercise with slowly forced expiration	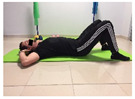	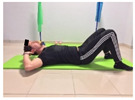	1
Oblique abdominal exercise with slowly forced expiration	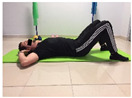	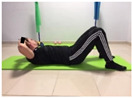	2
Roll onto the back, keeping knees to chest	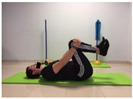	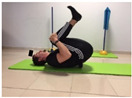	1
Abdominal exercise raising opposite arm/leg with slowly forced expiration	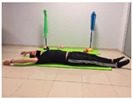	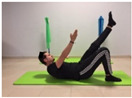	2
Roll from one sacroiliac to the other, keeping knees to chest	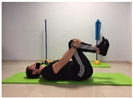	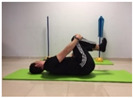	1
Abdominal exercise with slowly forced expiration with leg raise to 45 degrees	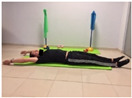	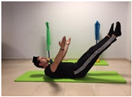	2
Alternative leg lifts with spine elongation	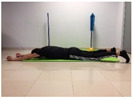	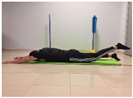	2
Alternative arm/leg lifts with spine elongation	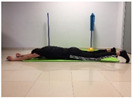	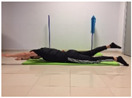	2
Simultaneous arm/leg lifts with spine elongation	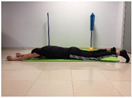	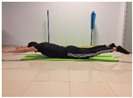	2

**Table 3:** Baseline of the studied variables.

Variable	ALL (*n* = 56)		CG (*n* = 26)		EG (*n* = 30)		*p*-Value
	*X* ± SD	Median	*X* ± SD	Median	*X* ± SD	Median	
Age (Years)	46.7 ± 10.5	50	46.1 ± 10.8	49.5	46.7 ± 9.3	47.5	0.82
Weight (Kg)	66.2 ± 8.8	57.7	61.6 ± 8.7	58.7	59.1 ± 8.9	56	0.29
Height (cm)	161.1 ± 9.2	158.6	163.2 ± 9.4	160.5	159.3 ± 8.8	157	0.12
BMI (Kg/m^2^)	23.1 ± 1.2	23.2	23 ± 1.3	23.3	23.2 ± 1.2	23.1	0.72
PCT	27.8 ± 3.6	28	26.7 ± 3	26	27.4 ± 3.9	27	0.44
BST	109.1 ± 13.6	110	107 ± 10.4	110	106 ± 14.6	110	0.77
MST	6.7 ± 0.5	6.7	6.6 ± 0.4	6.6	6.6 ± 0.5	6.7	0.91
TTT	0.8 ± 4.9	2	0 ± 4.8	0	−0.7 ± 4.5	−1.2	0.58
fSF-36	58.6 ± 4.7	57.9	58.3 ± 3.8	58.3	58.4 ± 5.2	57.9	0.97
pSF-36	57.5 ± 3.6	57.6	56.8 ± 3.7	57.9	58 ± 3.6	57.2	0.25

CG: Control group; EG: Experimental group; SD: Standard deviation; BMI: Body mass index; PCT: Partial curl-up test; BST: Biering Sorensen Test; MST: Modified Schöber Test; TTT: Toe Touch Test; fSF-36: physical dimension of the survey SF-36; pSf-36: psychosocial dimension of the survey SF-36.

**Table 4:** Inferential statistics of the 2 × 2 ANOVA test and effect sizes.

Variable	Group	Pre-Test		Post-Test		Group	M	Group × M	Cohen’s d
		Mean	95% CI	Mean	95% CI	*p*-Value			
PCT	CG	26.7	[25.5–27.9]	26.8	[25.5–28]	0.01	0.03	0.04	0.75
	EG	27.4	[26–28.9]	30.2	[28.9–31.4]				
BST	CG	107	[102.8–111.2]	105.6	[101.3–109.8]	0.03	0.048	0.01	0.77
	EG	106	[100.5–111.5]	117.2	[111.7–122.6]				
MST	CG	6.6	[6.4–6.8]	6.6	[6.4–6.8]	0.01	0.03	0.02	0.82
	EG	6.6	[6.4–6.8]	7	[6.8–7.3]				
TTT	CG	0	[−2–2]	−0.1	[−2–1.6]	0.07	0.01	0.01	1.02
	EG	−0.7	[−2.4–1]	3.8	[2.2–5.5]				
fSF-36	CG	58.3	[56.8–59.9]	57.9	[55.8–60]	0.27	0.62	0.3	0.28
	EG	58.4	[56.5–60.3]	59.8	[58.1–61.5]				
pSF-36	CG	56.8	[55.3–58.4]	56.5	[55.2–57.8]	0.02	0.89	0.51	0.15
	EG	58	[56.7–59.3]	58.5	[57.2–59.9]				

CI: Confidence Interval; PCT: Partial curl-up test; BST: Biering Sorensen test; MST: Modified Schöber Test; TT: Toe Touch Test; fSF-36: physical dimension of the survey SF-36; pSf-36: psychosocial dimension of the survey SF-36.

## Data Availability

The datasets generated during and analysed during the current study are available from the aim author or the corresponding author on reasonable request.
